# The Role of Artificial Intelligence-Powered Imaging in Cerebrovascular Accident Detection

**DOI:** 10.7759/cureus.59768

**Published:** 2024-05-06

**Authors:** Natasha Hastings, Dany Samuel, Aariz N Ansari, Purvi Kaurani, Jenkin Winston J, Vaibhav S Bhandary, Prabin Gautam, Afsal Latheef Tayyil Purayil, Taimur Hassan, Mummareddi Dinesh Eshwar, Bala Sai Teja Nuthalapati, Jeevan Kumar Pothuri, Noor Ali

**Affiliations:** 1 School of Medicine, St. George's University School of Medicine, St. George's, GRD; 2 Radiology, Medical University of Varna, Varna, BGR; 3 Internal Medicine, Era's Lucknow Medical College and Hospital, Lucknow, IND; 4 Neurology, Dnyandeo Yashwantrao (DY) Patil University School of Medicine, Navi Mumbai, IND; 5 Electronics and Communication Engineering, Karunya Institute of Technology and Sciences, Coimbatore, IND; 6 Radiology, Srinivas Institute of Medical Sciences and Research Center, Mangaluru, IND; 7 Emergency Medicine, Kettering General Hospital, Kettering, GBR; 8 Surgery, Barking Havering Redbridge University Hospitals NHS Trust, London, GBR; 9 Neurosurgery, Houston Methodist Neurological Institute, Houston, USA; 10 General Medicine, Mahavir Institute of Medical Sciences, Vikarabad, IND; 11 Internal Medicine, Maheshwara Medical College, Patancheru, IND; 12 Radiology, Government Medical College Suryapet, Suryapet, IND; 13 Medicine and Surgery, Dubai Medical College, Dubai, ARE

**Keywords:** stroke imaging modalities, artificial intelligence-assisted imaging, artificial intelligence in radiology, cerebrovascular accident detection, tele-stroke

## Abstract

Cerebrovascular accidents (CVAs) often occur suddenly and abruptly, leaving patients with long-lasting disabilities that place a huge emotional and economic burden on everyone involved. CVAs result when emboli or thrombi travel to the brain and impede blood flow; the subsequent lack of oxygen supply leads to ischemia and eventually tissue infarction. The most important factor determining the prognosis of CVA patients is time, specifically the time from the onset of disease to treatment. Artificial intelligence (AI)-assisted neuroimaging alleviates the time constraints of analysis faced using traditional diagnostic imaging modalities, thus shortening the time from diagnosis to treatment. Numerous recent studies support the increased accuracy and processing capabilities of AI-assisted imaging modalities. However, the learning curve is steep, and huge barriers still exist preventing a full-scale implementation of this technology. Thus, the potential for AI to revolutionize medicine and healthcare delivery demands attention. This paper aims to elucidate the progress of AI-powered imaging in CVA diagnosis while considering traditional imaging techniques and suggesting methods to overcome adoption barriers in the hope that AI-assisted neuroimaging will be considered normal practice in the near future. There are multiple modalities for AI neuroimaging, all of which require collecting sufficient data to establish inclusive, accurate, and uniform detection platforms. Future efforts must focus on developing methods for data harmonization and standardization. Furthermore, transparency in the explainability of these technologies needs to be established to facilitate trust between physicians and AI-powered technology. This necessitates considerable resources, both financial and expertise wise which are not available everywhere.

## Introduction and background

Stroke is the primary cause of disability worldwide and is responsible for 11.6% of all deaths [1]. With approximately 795,000 Americans experiencing cerebrovascular accidents (CVAs) annually, the economic burden exceeds $56.5 billion according to the last report in 2018/2019 [2]. More than two-thirds of these stroke incidents are ischemic, arising from thrombosis due to damaged atherosclerotic plaques or secondary emboli from the heart [3]. Strokes, along with transient ischemic attacks (TIAs), are the most common clinical manifestations of CVAs, a term that refers to a wide range of acute pathological changes due to cerebral vascular damage resulting in neurological deficits reflective of the location of the ischemia or infarction. The extent of these acute vascular pathologic changes encompasses disorders arising from vascular endothelial damage, thromboembolism leading to vessel occlusion, endothelial stenosis leading to blockage of cerebral circulatory circuits, formation of arterial aneurysms, and alteration of vascular permeability. Other noteworthy presentations of CVAs include subarachnoid hemorrhages, arterial dissections, vascular dementia, cerebral arteriovenous shunts, and vascular diseases such as vasculitis and cerebral venous thrombosis [1]. The disabilities associated with CVAs may be acute or chronic and include both motor and sensory deficits that typically clinically present as motor and/or sensory loss. The negative impact on performance in daily activities can be quantified by calculating the Disability-Adjusted Life Years (DALY). DALY refers to the years of life that have been impacted by a disability due to the occurrence of an event, in this case, a CVA. The most prevalent disabling condition after a CVA is post-stroke cognitive impairment and dementia [4]. Interestingly, multiple clinical studies conducted on working populations have noted that work disability (sick absences) might be present in individuals even before the occurrence of their first CVA, almost four years prior [5].

The severity of the consequences following a CVA necessitates early detection and treatment. Hence, a fast-tracked, highly customized route of management is the international recommendation of the World Stroke Organization and aims to reduce morbidity and mortality associated with CVAs [3]. Imaging is the cornerstone of CVA diagnosis, and in this golden age of artificial intelligence (AI), the rapid advent of AI-based applications in diagnosis, prognosis, and management of neurological pathologies is not only rightly justified but also the need of the hour. Furthermore, the USFDA recognizes the emergent 40% rise of AI in healthcare annually, potentially reducing public healthcare costs by USD 150 billion by 2026 [6].

Present treatments, reliant on timely reperfusion and advanced neuroimaging, introduce new challenges. Current clinical trials are incorporating more advanced neuroimaging techniques to define treatment standards, leading to heightened economic and logistical challenges within the healthcare system. The integration of sophisticated imaging modalities, such as MRI and CT, has become pivotal in treatment decisions, culminating in improved patient care. However, this dependence on neuroimaging has given rise to issues of limited accessibility, shortage of specialists, and inter-observer variability especially in low/middle-income countries [7,8]. The urgency of CVA treatment, where every minute lost results in substantial neural damage, underscores the need for swift and precise interpretation of neuroimaging data. An increasing drive supports the adoption of AI methods to optimize workflow, enhance diagnostic and treatment processes, and augment the significance of quantitative imaging methodologies [9]. The implementation of machine learning (ML) in CVAs emerges as a paradigm shift. ML algorithms offer significant contributions, from early identification of imaging diagnostic findings, estimating time of onset, and lesion segmentation to predicting complications and patient outcomes after treatment thus not only promising to improve patient care by expediting diagnosis and treatment planning but also holds the key to unlocking deeper insights into neurological conditions [10-13]. These applications address the limitations of current practices, providing a promising outcome for diagnosis and for overcoming challenges in CVA diagnosis and treatment. As the healthcare system continues to evolve, the convergence of cutting-edge technologies and AI presents the opportunity for advancement, poised to revolutionize neuroradiology and work through the complexities of CVAs to enhance patient outcomes. In this review, an insight into the role of AI in emergent brain imaging is provided for improving the detection of CVAs and in tandem aiming to enhance diagnostic precision in response to real-world challenges.

**Figure 1:**
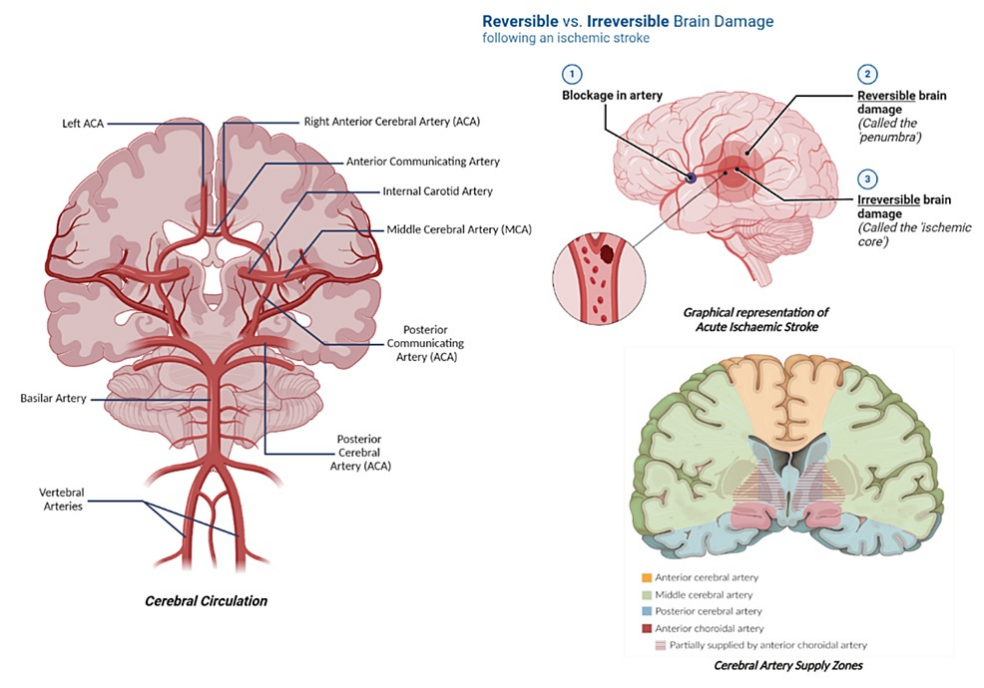
Circle of Willis and its branches. Created with Biorender.com.

Strokes can be classified into hemorrhagic and ischemic strokes. Etiologies of ischemic strokes include thromboembolism and hemorrhagic shock. However, hemorrhagic strokes occur due to severe bleeding due to arteriovenous (AV) malformation, aneurysmal rupture, a tumor, infection, vasculitis, or trauma. Distinguishing between the two types is paramount as management drastically differs. Thrombolytics are used to treat ischemic strokes; meanwhile, they worsen hemorrhagic strokes. Most of the brain's arterial supply is dependent on the perfusion supplied by the Circle of Wills [14]. This circulatory flow starts from the ascending internal carotid arteries and the vertebral arteries which perfuse the anteromedial and posteroinferior aspects of the brain respectively, as represented in Figure 1.

**Figure 2:**
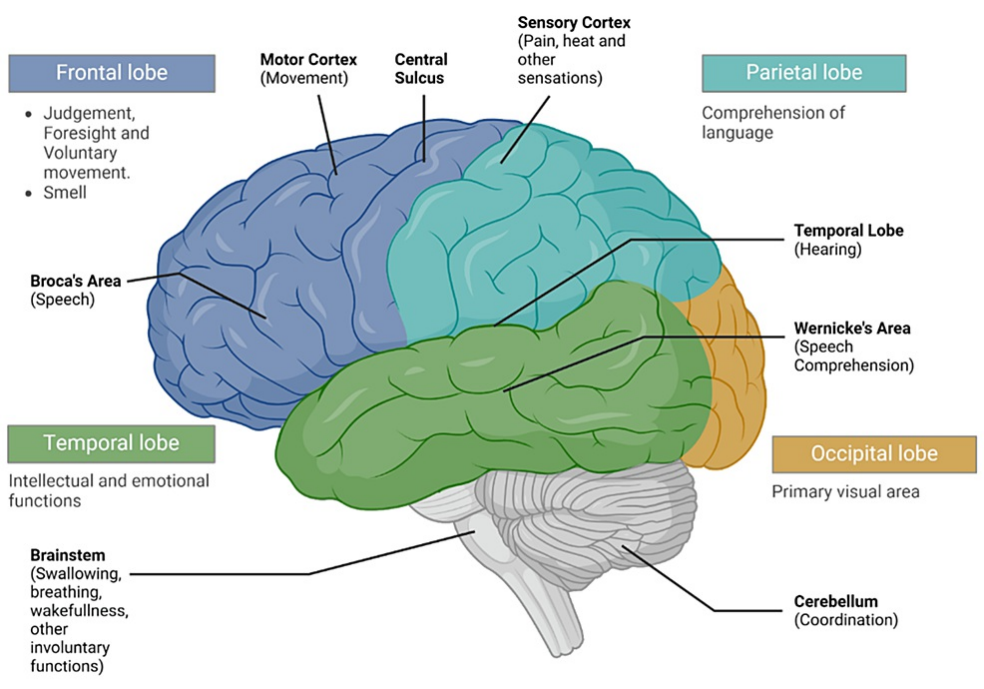
Motor and sensory regions of the cerebral cortex. Created with Biorender.com.

The anteromedial circuits comprise the anterior cerebral artery (ACA), which supplies the frontal, parietal, and cingulate cortex, the middle cerebral artery (MCA) that perfuses much of the lateral surface of the cerebral hemispheres and temporal lobe, and the posterior cerebral artery (PCA) that supplies the occipital lobe and various smaller communicating arteries. The posteroinferior circuit, or more correctly, the vertebrobasilar circulation is responsible for adequate perfusion of the cerebellum, brain stem, and occipital lobe. Thus, a stroke affecting any of these arteries would lead to ischemia or infarction in the respective functional areas, as depicted in Figure 2.

Figure 2 represents the functional associations of the brain depicting areas most prone to CVAs. ACA and MCA lesions are the commonest, wherein depending on the territory supplied by the branch, the clinical presentations vary [3]. Besides these, important stroke syndromes include but are not limited to Weber Syndrome (medial midbrain infarct), Benedikt Syndrome (ventral midbrain infarct), Nothnagel Syndrome (superior cerebellar lesion), Wallenberg Disorder (lateral medullary syndrome), Dejerine Syndrome (medial medullary infarct), and Locked-in Syndrome (pseudocoma due to basilar circulation infarct). In practice, it is very difficult to pinpoint the location of ischemia or infarction without radiological assistance, and this is where AI use can improve neuroimaging [15].

AI, which is a technology that enables machines to mimic human intellect, can be further dissected into its various subsets and learning techniques. ML is the primary subset of AI which uses large datasets and statistical methods to teach machines to recognize patterns, specifically patterns of abnormal neuro-pathological changes. While deep learning forms the basis of neural networks and enables three types of learning. Firstly, it enables supervised learning wherein the machine learns from preset information sets and learns to create patterns. Secondly, unsupervised learning results in identification amongst relatively larger, heterogeneous, random datasets. Finally, reinforcement learning is a positive and negative feedback-based two-way responsive learning technique. These learning methods are employed on large datasets of neuroradiological modalities to be able to rapidly recognize and accurately diagnose various CVAs. 

The use of AI-based computing methods to evaluate and screen neuroradiological images falls under the domain of the interdisciplinary research area of computational neuroimaging (CN). Within the last couple of decades, the rise in the number of neuroimaging pipelines like FastSurfer, FreeSurfer, and Insight Toolkit (ITK) is evidence of the rapid growth of CN technologies [16]. Furthermore, AI-based, FDA-approved applications have surfaced that can accurately detect and help manage CVAs; these include FastStroke (computed tomography angiography (CTA) and computed tomography perfusion (CTP)-based stroke detection) and Neuro-AI Algorithm (analyzes changes in brain perfusion) [6]. Other commercially available CN platforms include Brainomix (deep learning-based CTA analysis) and RapidAI (ML-based non-contrast CT analysis) [10]. These CN tools potentially lay the base for the development of further four-dimensional model-based, tailored management techniques for the reduction of morbidity and mortality from CVAs.

## Review

AI-assisted neuroimaging algorithms and methods

The biomarkers of CVAs can be captured through images, blood sample reports, coagulation markers, and other sensor signals. In medical image analysis, the area of lesions or hemorrhages is segregated with the help of quantitative features or descriptors from positron emission tomography (PET), computerized tomography (CT), or electroencephalogram (MRI) images. The features extracted from the region of interest can be given for the classifier model to predict the occurrence of stroke. A similar approach can be done through exploratory data analysis of clinical data like medical history, vital signs, symptoms, and laboratory results. Combining medical images and clinical reports helps to make better-informed predictions. With the help of AI algorithms, both behavioral changes detected through ambient sensors present in smart homes and signals captured through wearable devices can indicate the risk of stroke in patients.

ML and deep learning algorithms work on data captured from patients through different modalities like electrocardiogram (ECG), photoplethysmography (PPG), electroencephalogram (EEG), foot pressure, gait, MRI, CT scans, PET scans, and electronic medical records. The computational requirements of AI algorithms on these datasets will demand processors that consume significant amounts of energy and huge processing power. However, it is not feasible to have such embedded systems that do this in real time. Hence, it is compelling to develop ultra-low-power AI chips that can run inferences of AI algorithms at a faster rate and consume low energy [17]. Approaches to training AI models vary between supervised learning, unsupervised learning, transfer learning, and reinforcement learning. The designed model must be trained with the openly available dataset and tested to validate its performance. Common metrics like accuracy, precision, sensitivity, and specificity help in identifying the reliability of the AI model developed. The available open datasets help in validating the robustness of the algorithms developed. A few open datasets that can be used for detecting CVAs are given in Table 1.

**Table 1:** Open datasets for cerebrovascular accident detection. Created with Microsoft Word MRI: Magnetic resonance imaging; CT: computed tomography; EHR: electronic health record (EHR) Source: [18-22]

Dataset	Signal Modality	Dataset Size
Anatomical Tracings of Lesions After Stroke (ATLAS)	MRI	955
Ischemic Stroke Lesion Segmentation (ISLES) 2017	MRI	55
Intracranial Hemorrhage Detection Challenge 2019 by the Radiological Society of North America (RSNA)	CT	25000
Stroke Initiative for Gait Data Evaluation (STRIDE)	Gait	55
McKinsey & Company	EHR	548

The development of soft computing algorithms has shown promising results in predicting CVAs in patients irrespective of age, gender, blood pressure, diabetes, obesity, and smoking. Non-linear Hebbian learning from fuzzy cognitive maps can help in evaluating the risk rate of CVAs [23]. In addition, statistical methods like the random forest, K-nearest neighbor, logistic regression, AdaBoost classifier, gradient boosting, and nearest centroid are common methods that can yield good results for CVA prediction [24]. The severity of the CVA is based on the size and location of the lesion. Deep learning models can detect and segment these lesions from neuroimages, although an enormous amount of data is required for both testing and training. Methods like augmentation help in extrapolating more duplicate data samples from the original set obtained from clinical trials. U-Net architectures help in fusing multiscale information and segmenting the lesions from the magnetic resonance images to explore their spatio-temporal features [25]. End-to-end deep learning architecture like fully convolutional networks also can preserve spatial information and improve segmentation accuracy [26]. To further improve the segmentation, a dual attention gate is introduced into the U-Net architecture to intuitively access the features of lesions and segment them more accurately [27]. However, in the case of microbleeds that cannot be accurately delineated from the boundary region of a lesion, a two-stage deep learning approach is used, where the first stage utilizes a You Only Look Once (YOLO) model to detect the microbleeds and three-dimensional convolutional neural network (3D-CNN) approach to reduce the false alarms in the second detection phase [28].

Transfer learning trains existing architecture to a more defined use case. Pre-trained networks like ResNet-50, Visual Geometric Group (VGG-16), and GoogleNet are residual networks developed for image classification. However, modifying the input and output layer and training with brain images of CVA patients makes it trained to predict the brain images having infarcts. These methods can largely assist radiologists. Hybrid architectures interlink more than one AI model to aid in the diagnosis of CVAs. A convolutional network can link with a support vector machine (SVM)-based classifier for CVA prediction [29]. The convolutional network can extrude the textural features in the input image. The feature set when given as input to a rational classifier like SVM can predict with more accuracy. Hybrid approaches and transfer learning give more possibilities for building robust networks for diagnosing CVAs. It also significantly reduces the time required for building models from scratch. In elderly patients affected by a CVA, timely detection and intervention are critical for reducing morbidity and mortality rates. The computer-aided diagnosis may delay the treatment phase. Point-of-care testing devices need to be employed to rapidly assess the biomarkers. Hence, lightweight data like information collected from ECG electrodes placed in the thoracic region and PPG sensors from the middle finger can be used to predict CVAs while walking [30]. Ensemble algorithms can be used to leverage the benefit of multiple AI algorithms as they combine multiple models and make predictions for more robust decisions. For example, an AI ensemble combines the results of both convolutional neural network (CNN) and long short-term memory (LSTM) to predict the onset of a CVA [31]. Another way to predict a CVA is by EMG data gathered from bicep and calf muscle sensors that use myoelectric biomarkers. Sequential network models like long short-term memory have the property of memory retention and can predict the occurrence of CVA and can be used to alert medical staff in hospitals [32]. The international measurement units by wearable sensor technologies also help in grabbing the gait information from the patients affected with CVA. The abnormalities in gait like the drop-foot gait, the circumduction gait, the hip hiking gait, and the back knee gait help in understanding the muscular coordination of post-CVA patients. A deep neural network (DNN) model can analyze the gait patterns and locate the CVA [33]. Hybrid approaches like combining CNN features with long short-term memory prediction can also yield improved prediction results [34].

Traditional imaging challenges in detecting CVAs

The principal objective in imaging patients presenting with acute stroke symptoms is to differentiate between hemorrhagic and ischemic strokes [35]. Non-contrast CT (NCCT) is effective in excluding intra-cranial hemorrhage (ICH) before intravenous tissue plasminogen activator (tPA) [36]. Diffusion-weighted imaging (DWI) and gradient echo sequences have comparable accuracy to NCCT in identifying hyperacute intraparenchymal hemorrhage within a six-hour onset [35]. CTA is beneficial if either ischemic or hemorrhagic stroke is suspected since it visualizes intracranial arteries to identify and assess the potential underlying cause as well as any vascular malformations or aneurysms contributing to the event [37]. For individuals awakening with a stroke or presenting with an uncertain onset time beyond 4.5 hours from baseline or the last known well state, employing MRI to detect diffusion-positive and fluid-attenuated inversion recovery (FLAIR)-negative lesions are valuable [36]. This helps identify patients who may benefit from intravenous alteplase or tenecteplase within the crucial 4.5-hour window from stroke symptom recognition. Additionally, the utilization of CTA paired with computed tomography perfusion (CTP) or magnetic resonance angiography (MRA) combined with diffusion-weighted MRI (DW-MRI), with or without perfusion, is crucial for assessing and selecting candidates for mechanical thrombectomy within the extended timeframe of 6 to 24 hours after the last known well state [36]. 

NCCT imaging often lacks sensitivity for detecting posterior fossa infarcts and early ischemic changes. CTP images come with challenges, including limited interobserver reliability, variations in defining infarct core and penumbra thresholds, relatively high ionizing radiation doses resulting from contrast bolus tracking, and artifacts related to motion & bolus [38]. The effectiveness of CTA also relies on precise timing, meticulous technical planning, and sufficient cardiac output & flow through vasculatures [39]. Any disruption in these conditions can lead to suboptimal images, diminishing the diagnostic yield of the procedure. When NCCT is inconclusive, MRI emerges as the preferred imaging modality to rule out posterior fossa infarction [40]. Despite MRI having higher sensitivity and specificity in the diagnosis of acute ischemic infarction in the first few hours after onset than CT, MRI is more time-consuming and less available than CT [41]. MRI also has drawbacks in patients with metallic foreign bodies like old cerebral artery aneurysm clips, certain pacemakers, cardiac prostheses, certain ear implants, catheters with metallic components, cerebral artery aneurysm clips, and those deemed medically unstable [41]. Additionally, MRI is more susceptible to motion artifacts, posing a notable limitation in patients experiencing acute neurological changes. The subtle nature of infarction signs in acute stroke and the intrinsic variability in human assessments pose a delay in the precise interpretation of images, such as NCCT scans for patients with acute ischemic or posterior fossa strokes. This necessitates waiting for the radiologist's report or consultation with neurologists for decision-making, things not readily available, especially in smaller hospitals.

AI-assisted neuroimaging versus traditional imaging modalities

Information obtained from imaging studies is critical for successful stroke management. Both NCCT and MRI are important in differentiating ischemic versus hemorrhagic strokes, a determination that can be difficult to achieve by clinical features alone. The detection of irreversibly damaged tissue is made easier by hypodensity in CT or DWI hyperintensity on MRI, where the latter has been shown to have better sensitivity than MR sequences, especially during acute circumstances [42]. Angiographic and perfusion imaging sequences contribute to the diagnosis of large vessel occlusion and, thus, help in patient selection for endovascular intervention. The DWI-DWI-FLAIR mismatch offers important information for cases where the onset time is unknown, such as wake-up strokes. Furthermore, stroke imaging provides predictive value where current approaches seek to portray the sequelae of successful reperfusion or continued extensive vessel obstruction in a short-term manner. An essential aspect of stroke imaging is the need for immediate interventions because greater urgency correlates with better outcomes [43]. However, several stages of the stroke-imaging triage route depend on radiologists and neurologists, posing a time constraint. The given tasks require a high degree of specialized expertise to be performed, which is not always accessible everywhere. Thus, the need for education and implementation of these automated CVA imaging and evaluation techniques is paramount, especially in areas of the world that lack high neurologist expertise. 

Despite AI's potential advantages with time lag, reservations have been made about the lengthy process of some advanced imaging modalities such as CT perfusion or MRI, which can extend both acquisition and post-processing times compared to NCCT and CT angiography. Although MRI stroke protocols usually require a scan time of less than 10 minutes, detailed screening and patient transfer time are implemented which requires continuing efforts to decrease overall scan time. AI-based approaches increase the speed of imaging using deep learning algorithms, especially convolutional neural networks, and improve image quality by reducing image noise - the variation of color brightness- and extracting prominent features from input images. They also administer lower radiation doses by establishing and focusing on a region of interest way faster than human operators [44]. Deep convolutional neural networks also apply to other MR sequences including quantitative susceptibility mapping that might detect brain hemorrhage and calcification [45]. AI techniques can solve the problems with gadolinium deposition by lowering the amount of contrast agent required. Moreover, convolutional neural networks reduce the radiation dosage, especially in CT scans where issues such as high-dose procedures like CT perfusion imaging are addressed [45,46]. An innovative application of deep learning is in predicting imaging biomarkers that are hard to acquire, including cerebral blood flow. The to-be-explored paradigm has the potential to optimize image processing irrespective of clinical settings [47]. Typically, perfusion parameters are used in the process of predicting functional outcomes for both infarcted (core) and at-risk tissue [42]. On the other hand, AI offers an opportunity to directly predict these outcomes from source perfusion images themselves. 

While AI has shown promising results in stroke imaging, there are still several advantages of the traditional approach that are worth considering. One of the key advantages is the extensive experience and expertise of human radiologists and healthcare professionals. Their ability to interpret complex imaging results, considering nuances and details that may not be easily discernible by AI systems, is invaluable in the accurate diagnosis and treatment of strokes. Additionally, the traditional approach allows for a more personalized and patient-centric analysis. Healthcare providers can consider a patient's medical history, individual risk factors, and specific symptoms in conjunction with imaging results to make well-informed decisions [48]. When assessing the diagnostic accuracy and clinical outcomes of commercially available stroke imaging AI tools like Brainomix, RapidAI, and Viz.ai, studies with large test datasets revealed ischemia detection sensitivities ranging from 44% to 83% and specificities from 57% to 93% [48]. Similarly, the assessment of large vessel occlusions (LVOs) also demonstrated variations in diagnostic accuracy across the studies reviewed. This highlights the need for further investigation and clinical validation of these AI technologies in stroke imaging to fully understand their potential benefits and limitations in identifying key prognostic features of acute ischemic stroke [48].

Notable studies and breakthroughs in AI-assisted neuroimaging

A multitude of studies have been conducted concerning AI and its potential to enhance the early detection of CVAs. Oftentimes, there have been investigations that show promise for the role of AI in the diagnosis and triage of patients with CVAs, especially those relating to LVOs, but research continues to be limited and there is not enough data to support its integration into clinical settings [49]. Supportive data is coming in waves with continuous successful clinical trials regarding the implementation of AI in early stroke detection and management. The bias remains in studies because of single-center populations under investigation as well as small patient numbers, making it difficult to apply the results to broader patient populations. Viz.ai, a leader in medical AI technology, sought to address these biases by conducting a large multicenter study. The study was conducted in partnership with TeleSpecialists LLC, across 17 states in 146 facilities with 14,116 patients [50]. Two cohorts were examined, the Viz cohort of 8,557 patients in 76 hospitals and the non-AI cohort of 5,559 patients in 90 hospitals. In the cohort that used Viz.ai technology, the median arrival time (to the hospital) to neuro-interventionalist notification times were reduced by a whopping 39.5 minutes. With respect to the 1.9 million neurons, 13.8 billion synapses, and 12 miles of axonal fibers lost each minute in which a stroke is untreated, that equates to 75.05 million neurons, 545.1 billion synapses, and 474 miles of axonal fibers that can potentially be saved with the intervention of reliable and accurate AI technology [50].

Clinically relevant reductions in endovascular stroke therapy (EVT) in LVOs were recorded in a cluster randomized stepped-wedge clinical trial in Houston, Texas that assessed the primary outcomes of patients needing acute stroke triaging [51]. This study was conducted in four comprehensive stroke centers with 243 participants via enabling AI-automated CT angiogram coupled with secure messaging. This system resulted in a 9.8-minute reduction (96% CI, -16.9 to -2.6) in time from CT scan and initiation of EVT, further reducing the length of hospital stay and ultimately causing an 18% decrease in mortality [51]. With those previous numbers in mind, a 9.8-minute reduction is equivalent to 18.62 million neurons, 135.24 billion synapses, and 117.6 miles of axonal fibers potentially spared, per patient, due to the integration of AI and closed-loop messaging systems. While the cohort in this trial was significantly smaller, the results consistently supported the implementation of AI, with streamlined communication systems in place to improve clinical workflow as well as clinical outcomes in patients with CVAs.

A study in China used deep learning-based DW-MRI imaging to aid in the diagnosis of early cerebral infarctions of 210 patients. DWI is currently the standard used in clinical diagnosis of ischemic brain infarctions, the physiological process resultant of a CVA. DWI was paired with a co-administered algorithm, higher-order singular value decomposition (HOSVD) denoising algorithm (GL-HOSVD), to denoise the images of subjects and results were then compared to DWI alone and in addition to other algorithms [52]. In this study, a control group of 105 patients was subject to DWI alone while the observed group of 105 patients had DWI imaging with the HOSVD denoising algorithm (GL-HOSVD), and each method was assessed for its sensitivity, specificity, accuracy, and consistency in identification of ischemic pneumbra (IP), reversibly injured tissue surrounding an ischemic core. With statistically superior results, 87.6%, 81.25%, and 87.62% respectively, in the observation group, a statistically considerable (P < 0.05) comparison to 57.78%, 53.33%, and 57.14% of the control group. This study reinforces the significant role of AI in the timely diagnosis and management of CVA patients [52].

Challenges associated with adopting AI-assisted neuroimaging 

Despite the various potential benefits of AI neuroimaging, multiple technological, operational, economic, social, and ethical challenges warrant consideration. There is a need for extensive training and validation of AI algorithms using large datasets. The accuracy and reliability of AI systems in detecting arterial occlusions and other stroke imaging abnormalities rely heavily on the quality and quantity of data used for their development. This process can be time-consuming and resource intensive. Moreover, while the diagnostic accuracy of e-CTA (product name) for identifying acute arterial abnormalities was reported to be around 72-76%, it is crucial to understand that this still falls short of the expertise provided by human radiologists. AI systems may encounter challenges in discerning subtle details and variations in imaging results, which could impact the accuracy of diagnosis and subsequent clinical decisions. On a population level, the use of AI in stroke imaging presents the challenge of false positives and false negatives, which may have implications for patient outcomes. Based on the range of published sensitivity and specificity results for stroke feature detection by AI software, it is estimated that for every 100 patients assessed using this software ischemia was correctly detected in 44 to 83 patients suffering ischemic strokes but missed in 17 to 56 patients. In the absence of an ischemic stroke, ischemia was detected in 7 to 43 patients. In large vessel occlusion, occlusion was correctly detected in 80 to 96 patients but missed in 4 to 20 patients. Without LVO, occlusion was incorrectly detected in 2 to 10 patients. These findings highlight the potential for both false positives and false negatives in AI-based stroke imaging, which can impact patient outcomes [48].

The training datasets for ML may be inadequate or unverified from the electronic health records posing a discrepancy in data accuracy and validation creating a bias in the models [53]. One scenario of bias may occur when the training data set is focused and acquired from a certain demographic group, leaving others unavoidably under-represented. Therefore, models can be inherently biased towards a specific group. Another example is an algorithm created using data collected from black men aged 45-60 years yielding unreliable results when applied to black or white women [54]. 

A peculiar character of ML models is the ‘black box’. This characteristic perhaps raises the most difficult challenge in the deployment of ML in healthcare institutions. It can be described as an obstacle to the unrestricted demonstration of information to physicians, thereby raising issues of transparency in the algorithms. The lack of transparency reduces the authenticity of the models and therefore, builds untrustworthiness among those implementing it in a real-world scenario. ML approaches are based on predetermined engineered components, which reduces their applicability in situations such as cerebrovascular accidents, where symptoms and disease progression vary and evolve. This establishes a need for regular and continuous ‘real-time’ life updates to ML models to allow the incorporation of substantial, high-quality datasets that can be tried and tested across various patient demographics. Compared to ML, deep learning subsets of artificial intelligence are much more advanced and sophisticated, yet the interpretability of deep learning approaches is not entirely clear and understood. The lack of explainability of AI-operated models builds uncertainty regarding their free and fair application. Further, deep learning models are trained on retrospective data and smaller sample sizes [55]. This limited data variation fitted to the algorithms, not only produces poor and distorted results but also restricts its utility on larger patient groups. Both machine and deep learning models have overfitting issues. Overfitting happens when results are influenced by irrelevant miscellaneous variables and patient features that are not specific to the training data set on which the algorithm was developed [56].

Another major limitation of adopting these technologies involves the operational gap in the accessibility of relevant information which poses regulation and compatibility concerns among institutions. The integration of large numbers of multi-modal imaging data and clinical data from multiple centers and hospitals is crucial to creating a standardized, structured cloud system of storage. A uniform database allows less scope for error toward an efficient and unbiased automated detection of strokes across different centers. Besides the internal efficiency and security of an algorithm, there are risks to the external security of models. Computer-assisted tools can be hacked into and breached by cyberattacks. A breach of data can ruin the groundwork on which algorithms and models are manufactured. Patient information can be intentionally tampered with, resulting in significant changes in the recommendations of a well-trained model [57].

AI can be an easy distraction in an overwhelming workplace, the array of machine-enabled decision support tools already available could induce technological dependence for clinicians and interfere with their autonomy. Hesitation and skepticism regarding the use of advanced techniques will persist amongst medical professionals if they are unable to understand the justification behind the AI-operated conclusion, which is a challenge as most models lack interpretability. Physicians must comprehend the rationale behind a suspected stroke diagnosed by an automated computer-assisted screening tool i.e ‘StrokeAlert’ to confidently adhere to the proposed diagnosis and proceed with the necessary treatment [58].

The potential benefits of implementing AI in stroke imaging are balanced by the significant costs associated with acquiring and utilizing these technologies. The discussion of costs must consider the realistic value and impact of AI tools in clinical practice. It is essential to consider the financial implications of integrating AI tools into healthcare settings, especially in the context of stroke imaging. Currently, one typical commercial AI software costs around US$47,868 for one hospital for one year in the United Kingdom, equivalent to about a third of a hospital consultant's salary. This raises important questions about the cost-effectiveness of such technologies, particularly when considering the limitations and challenges associated with their diagnostic accuracy and clinical outcomes. The substantial cost of AI tools in stroke imaging may seem unreasonable, especially when considering that these tools only identify a few features in one disease, should only be used by an experienced medic, and have a limited evidence base. It is crucial to weigh the potential benefits against the financial investment required, as well as the existing expertise and capabilities of healthcare professionals in traditional stroke imaging approaches [42]. Accessibility, constant funding, large data infrastructure, and specialized algorithm developers remain a challenge in developing countries and peripheral areas of developed countries. Furthermore, the existing racial, age, and gender prejudice in our society is translated into the functional core of AI, therefore widening the gap between the rich and the poor [56]. Extensive development of multi-ethnic algorithmic training datasets is paramount to creating an inclusive technology. 

Healthcare professionals are ethically bound to the Hippocratic oath and Health Insurance Portability and Accountability Act (HIPAA). Obtaining consent from patients to utilize their data in the training of AI subsets should be the foremost priority while establishing and adopting such models. Patients provide their information with confidence that they will be protected and taken care of. Therefore, it is not only the primary responsibility of physicians but also, of those involved in the development of the models, to safeguard the data from infringement. Additionally, the accountability of AI is still a debatable subject [35]. In situations where a treatment protocol based on the diagnosis of a well-trained AI model does not prove suitable for the patient or in cases where the algorithms produce erroneous predictions due to a malfunction, deciding whom to hold accountable will be challenging. The responsibility for clinical decisions, however, especially those with significant implications for patient care, ultimately rests with healthcare providers [57]. Thus, the utilization of AI in stroke imaging necessitates clear guidelines and regulations to ensure patient safety and quality of care. 

Call to action

Diligent efforts and strategic focus are needed to guide future advancements in AI neuroimaging. Future research must prioritize the development of interpretable AI models capable of explaining their reasoning, thereby bridging the gap between predictive power and transparency. According to the reviewed literature, AI algorithms exhibit varied sensitivity metrics, with an average of 68%. This suggests that some algorithms may fail to detect up to one-third of imaging findings. Common reasons for algorithmic failures include abnormalities in radiologic scans from existing CNS injuries, inadequate contrast, challenges in motion correction, and difficulties in evaluating the contrast column due to tortuous vessels [59]. Neuroimaging data quality and heterogeneity significantly impact AI model performance. To enhance the generalizability and robustness of AI models, future efforts must focus on developing methods for data harmonization and standardization.

To increase adoption, efforts must be made to improve the explainability of these technologies and clinical confidence. This can be achieved by enhancing transparent ML methods with algorithm optimization and utilization of deep learning methodologies. To increase clinician confidence and transparency, it is essential to utilize combinations of ML techniques to mitigate the tradeoff between interpretability and accuracy. While there is vast potential for the adoption of ML/AI in the clinical setting, strong efforts are yet to be made to improve the explainability of these technologies. These strides can only be made by enhancing transparent ML methods, algorithm optimization, and utilization of deep learning [60]. In addition, seamless integration into clinical workflow is crucial [61]. To achieve this, research should concentrate on developing user-friendly interfaces and decision support systems that seamlessly integrate AI into clinical practice. However, the use of automated systems in clinical decision-making for cerebrovascular neurosurgeons raises ethical concerns. While AI can enhance standardization and analyze more variables, it also leads to a high rate of false positives [62]. This underscores the importance of continuous improvement in AI algorithms and emphasizes that, while valuable, AI should complement rather than replace clinician judgment.

## Conclusions

A highly customized, expedited treatment protocol is required to reduce the increasing morbidity and mortality rates associated with CVAs. Given the intricacies of the pathological changes caused by CVAs, imaging continues to be the most indispensable approach for prompt detection and diagnosis. Widespread revolutionization of AI in neuroimaging is the need of the hour to reduce disabilities and bring a positive impact to those affected by CVAs. The shift toward the integration of AI stems from the challenges faced with traditional imaging methods. AI-powered neuroimaging can potentially accelerate the management of CVAs solving the issue of time constraints. In addition, it can serve as a compensatory measure to minimize the growing burden on healthcare professionals. However, despite AI transforming the outlook of neuroimaging, there are still several limitations that need to be addressed before AI can completely replace or be at par with the traditional approach. Cost and logistic challenges associated with these technologies must be carefully evaluated to determine their realistic value in enhancing patient care and clinical outcomes. Future research must focus on the development of standardized, affordable, and accessible models along the lines of ethical regulations to ensure the responsible deployment of AI in stroke imaging. Strategic efforts must be made to overcome adoption barriers, and these efforts should be monitored in a timely manner to track the growth of adoption. 
